# Anti-obesity activity of hen egg anti-lipase immunoglobulin yolk, a novel pancreatic lipase inhibitor

**DOI:** 10.1186/1743-7075-10-70

**Published:** 2013-12-09

**Authors:** Mai Hirose, Taishi Ando, Rahman Shofiqur, Kouji Umeda, Yoshikatsu Kodama, Sa Van Nguyen, Tsuyoshi Goto, Masaya Shimada, Satoshi Nagaoka

**Affiliations:** 1 Department of Applied Life Science, Faculty of Applied Biological Sciences, Gifu University, 1-1 Yanagido, Gifu 501-1193, Japan; 2 Immunology Research Institute in Gifu, EW Nutrition Japan, Gifu, Japan

**Keywords:** Obesity, IgY, Pancreatic lipase, Lipid, Mice

## Abstract

**Background:**

There is completely no report about both hen egg anti-lipase immunoglobulin yolk (IgY) and its anti-obesity action. Thus, we tried to isolate and characterize a novel anti-lipase immunoglobulin from hen egg yolk. Moreover, we investigated whether hen egg yolk anti-lipase IgY inhibits pancreatic lipase activity *in vitro*, and examined its ability to prevent obesity in a murine high fat diet-induced obesity model.

**Methods:**

We determined the inhibitory action of Anti-lipase IgY on lipase activity *in vitro*. We also focused our evaluation on the anti-obesity properties of Anti-lipase IgY in a murine high fat diet-induced obesity model.

**Results:**

Anti-lipase IgY blocked porcine lipase activity with an IC_50_ of 0.49 μM. Supplementing the high fat diet with only 0.2% (w/w) of Anti-lipase IgY for 35 days significantly decreased the weights of intraperitoneal adipose tissues, epididymal, mesenteric, retroperitoneal and perirenal adipose tissues, and the amounts of hepatic total lipid, triglyceride, and cholesterol. This was accompanied by a significant increase in the fecal excretion of triglyceride in the absence of diarrhea. Furthermore, Anti-lipase IgY treatment restored body weight gain to levels similar to mice fed with Control IgY.

**Conclusions:**

This study provides the first report of the development of anti-lipase IgY and the direct evidence that inhibition of pancreatic lipase using Anti-lipase IgY is an effective anti-obesity treatment due to the associated increase in fecal excretion of triglyceride.

## Finding

### Introduction

Obesity causes excess fat accumulation in several tissues in addition to white adipose tissue (WAT), such as other insulin-responsive organs including the skeletal muscle and liver; this predisposes individuals to the development of insulin resistance. Obesity is strongly associated with metabolic syndrome, which is characterized by the presence of insulin resistance, hypertension, and hyperlipidemia. Metabolic syndrome, which is closely linked to atherosclerosis, has become a major public health problem.

Several approaches for prevention and treatment of obesity have been reported [1]. Among these, both natural and synthetic pancreatic lipase inhibitors are effective in obesity prevention, likely due to their inhibition of intestinal lipid absorption. Indeed, a specific pancreatic inhibitor, Orlistat, has been used in the clinic for the prevention of obesity [2]. In animal studies, a major tea catechin, (−)-epigallocatechin-3-gallate [3] and polyphenol extracts in black tea [4] prevented high-fat diet-induced obesity via the inhibition of pancreatic lipase.

Another innovative candidate approach for pancreatic lipase inhibition is oral administration of anti-lipase immunoglobulin yolk (IgY) antibodies. There is completely no report about both hen egg yolk anti-lipase IgY and its anti-obesity action. Hens transfer immunoglobulin G (IgG) in blood into the egg yolk to provide acquired immunity to the offspring; this transferred IgG is termed IgY. The IgY technology offers several advantages over other methods of antibody production. For example, development and production of specific IgY can be achieved through collecting eggs from hens immunized with the target antigen, without the need for painful blood sampling and sacrifice of the animals.

The stability of IgY in pressure, heat, pH, trypsin, chymotrypsin, pepsin, and in the gastrointestinal tract has been well-documented [5]. IgY is relatively stable to pressure up to 4,000 kg per cm^2^. IgY is stable at temperature ranging between 30°C and 70°C. It was found that the activity range of IgY for pH was pH 3.5 ~11. The stability of IgY at pH 3 was increased in the presence of sorbitol [6]. IgY is quite resistant against trypsin and chymotrypsin inactivation, but degraded by pepsin [7]. Moreover, our group has investigated the *in vivo* passage and the efficacy of IgY in the gastrointestinal tract of piglets and calves. Our results indicated that IgY powder was transported as immunologically functional molecules from the stomach down to the small intestine of calves while retaining much of their original biological activity [8].

Exploiting the peculiarities of the avian immune system, it is possible to mass-produce specific IgY antibody against various antigens, since IgY levels are extremely high in egg yolk, and one hen lays 250–300 eggs per year. Indeed, other scientists as well as we have produced IgY antibodies against microorganisms such as cholera [9] and Candida [10] using this method. Furthermore, we have shown that oral passive immunization with IgY antibodies prevented and improved antigen-induced disease symptoms in animal studies [11,12].

Here, we describe the production of a novel anti-lipase specific IgY from egg yolk following immunization of hens with a porcine pancreatic lipase, a key enzyme required for lipid digestion. Since the oral passive IgY-based immunotherapeutic strategy outlined above was effective against bacteria, we predicted that our new approach would produce a selective inhibitor of pancreatic lipase activity. By extension, we hypothesized that this inhibitor may exert anti-obesity effects. Thus, in this study, we investigated whether hen egg yolk anti-lipase IgY inhibits pancreatic lipase activity *in vitro,* and examined its ability to prevent obesity in a murine high fat diet-induced obesity model.

## Methods and procedures

### Reagents

The enzyme substrates and reagents were as follows: triolein, taurocholate, colipase, and L-α-phosphatidylcholine were from Sigma (MO, USA). High pure porcine pancreatic lipase was obtained from Elastin Products Co (Owensville, MO, USA). The fresh porcine pancreas was obtained from the local slaughtering house in Gifu, Japan. Lipase purification procedure was based upon the method of Garner and Smith [13]. Anti-porcine pancreas lipase-specific IgY containing preparation (designated as Anti-lipase IgY) and control IgY preparation (designated as Control IgY) were kindly provided by EW Nutrition Japan. (Gifu, Japan).

### Anti-Lipase IgY preparation

Five-month-old White leghorn hens (Hyline W36; Japan Layer, Gifu) were immunized according to the method described by Yokoyama *et al.*[14]. Briefly, the vaccine was prepared by mixing 0.5 mg of purified lipase antigen with 0.5 ml emulsion oil containing 5% Arlacel 80 (Maine Biological Laboratories, Waterville, Me, USA) and hens were immunized by injecting 0.5 ml to each of the breast muscles. Six weeks after the initial immunization, a booster was given in the same manner. Eggs from the immunized hens were harvested daily and stocked at 4°C. Egg yolk was separated carefully from the albumin. The yolk was then pooled, homogenized, and filtrated. Partially purified specific IgY powder was prepared by ammonium sulfate precipitation [15], and freeze-dry. Control IgY powder was prepared from the egg of non-immunized hens by the same method.

### Assay to determine titer of anti-lipase specific IgY

The concentrations of both Anti-lipase IgY and Control IgY were determined using an enzyme-linked immunosorbent assay (ELISA) method as previously described [16].

### Lipase activity assay

The porcine pancreatic lipase activity was determined using the method of Tsujita *et al.*[17].

### Animals and diets

Male 6-week-old C57BL/6 J mice were purchased from Japan SLC (Hamamatsu, Japan). Mice were housed individually in standard plastic rodent cages, and placed in a room where the temperature was maintained at 22 ± 2.0°C with a 12-h light:dark cycle (lights on 0800–2000 h). All the mice consumed a commercial nonpurified MF (Mouse Flat) diet (Oriental, Yeast, Osaka, Japan) and tap water ad libitum for 4 days prior to their division into the following two weight-matched groups (n = 8: 8 mice per group): 0.2% (w/w) Control IgY-supplemented diet (CY) group, and 0.2% (w/w) Anti-lipase IgY-supplemented (AY) group. The compositions of the experimental diets are shown in Table 1. Mice were fed on these diets for 35 days. To determine the effective dose of Anti-lipase IgY in mice, we have done the preliminary experiment for 8 days. After this preliminary experiment, we have chosen 0.2% (w/w) of IgY in mouse study.

**Table 1:** Composition of experimental diets

**Components**	**CY**	**AY**
	g/kg	
Casein	286	286
Control IgY	2	-
Anti-lipase IgY	-	2
Corn starch	172	172
Sucrose	86	86
Cellulose	65	65
Soybean oil	40	40
Lard	300	300
Mineral^1^	35	35
Vitamine^2^	10	10
Choline chloride	4	4

^1^AIN-93G Mineral mixture.

^2^AIN-93G vitamin mixture.

Food intake and body weights of the mice were recorded daily during the feeding period. At the end of the experimental period, animals were anesthetized with ether after a 22-h fasting period. Livers and visceral fat pads were removed and weighed. The plasma, liver, and visceral fat pad samples were collected and stored at −80°C until analysis. Fecal collections (d 7–9) were used for determining fecal lipids. The care and experimental procedures were approved by the Animal Care and Use Committee of Gifu University.

### Biochemical analyses

Various lipid concentrations were determined using commercially available kits as follows: plasma, liver, and fecal triglyceride with Triglyceride E-test Wako (Wako Pure Chemical, Osaka, Japan) and plasma, liver, and fecal cholesterol with Cholesterol E-test Wako (Wako Pure Chemical, Osaka, Japan). Liver and fecal lipids were extracted by the method of Folch *et al*. [18], and total lipids were determined gravimetrically by the method of Nagaoka *et al*. [19]*.*

Plasma glucose levels were determined by the glucose CII test (Wako Pure Chemical, Osaka, Japan). Plasma insulin levels were measured with an ELISA kit (Morinaga Institute of Biological Science, Yokohama, Japan). Plasma TNF-α levels were measured with an ELISA kit (R&D Systems, Minneapolis, USA).

### Statistical analyses

All data presented in this study were normally distributed. Data were tested for normal distribution by the Kolmogorov-Smirnov normality test [20,21]. After Kolmogorov-Smirnov test, the statistical significance of differences was evaluated using the Student’s t-test [22].

## Results

### Titer of anti-lipase specific IgY

The titers of Anti-lipase IgY (AY) or Control IgY (CY) used for the mouse study were 25.72 ± 1.37 mg/g or 0 mg/g preparation, respectively.

### Inhibitory effect on lipase activity

**Figure 1:**
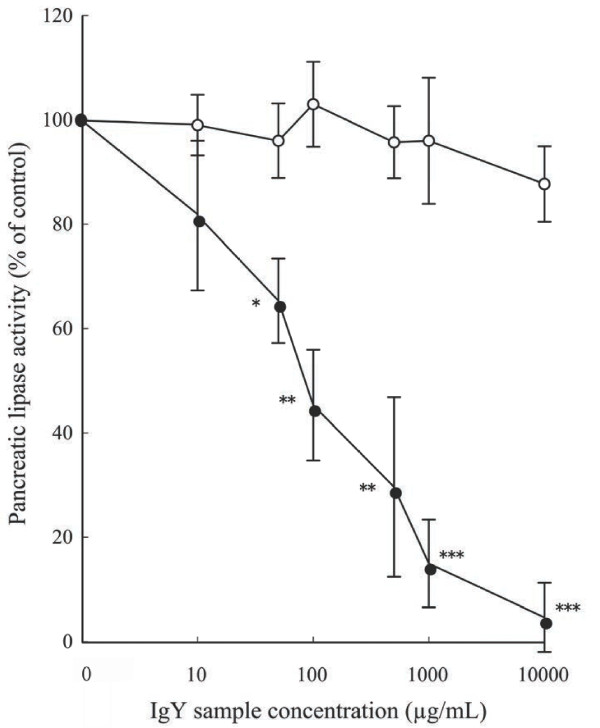
**Effects of increasing concentration of Control IgY (○) or Anti-lipase IgY (●) on pancreatic lipase activity.** The values are means ± SEM, n = 6. Differences from the Control IgY were calculated using Student’s t test (*p < 0.05, **p < 0.01, ***p < 0.001).

AY inhibited pancreatic lipase activity by 95.4〜18.3% at concentrations between 10,000〜10 μg/ml, whereas CY did not inhibit triolein hydrolysis by pancreatic lipase. The IC_50_ value of AY was 88 μg/ml (0.49 μM). Growth inhibition of pancreatic lipase occurred in a dose-responsive manner (Figure 1).

### Body weights, total food intake, fecal and tissue weights

Initial body weight, final body weight, body weight gains, total food intake, fecal and liver weights were all unaffected by dietary treatment (Table 2). The adipose tissue (epididymal, mesenteric, retroperitoneal and perirenal) weight was significantly lower in the AY group than that of the CY group.

**Table 2:** Effects of Control IgY (CY) and Anti-lipase IgY (AY) on physiological parameters

	**CY**	**AY**
Initial body weight (g)	27.57 ± 0.27	22.50 ± 0.38
Final body weight (g)	26.18 ± 0.75	25.47 ± 1.01
Body weight gain (g/35 d)	3.61 ± 0.88	2.97 ± 0.85
Total food intake (g/35 d)	99.27 ± 4.81	95.28 ± 2.31
Fecal dry weight (g/3d)	0.96 ± 0.03	0.91 ± 0.02
Tissue weight (mg/g B.W.)		
Liver weight	33.6 ± 0.64	34.7 ± 1.07
Epididymal WAT	26.8 ± 2.76	20.2 ± 0.40*
Mesenteric WAT	9.32 ± 0.94	6.57 ± 0.50*
Retroperitoneal WAT	14.0 ± 1.18	10.8 ±0.41*
Perirenal WAT	9.51 ± 1.31	6.32 ± 0.40*
Intraperitoneal WAT	59.6 ± 5.74	43.9 ± 0.73*
Tissue weight (mg)		
Liver weight	877.5 ± 22.6	877.6 ± 20.5
Epididymal WAT	709.9 ± 87.3	513.5 ± 20.4*
Mesenteric WAT	246.5 ± 29.9	169.0 ± 16.4*
Retroperitoneal WAT	332.0 ± 53.1	273.3 ± 13.0*
Perirenal WAT	251.6 ± 39.9	162.0 ± 13.5*
Intraperitoneal WAT	1573.4 ± 180.5	1117.8 ± 52.0*
Plasma parameters		
Triglyceride (mg/dl)	54.2 ± 3.6	44.6 ± 3.9
Cholesterol (mg/dl)	109.1 ± 3.9	104.6 ± 3.4
Glucose (mg/dl)	134.7 ± 7.1	137.1 ± 7.2
Insulin (ng/ml)	0.14 ± 0.02	0.14 ± 0.02
TNF-α (pg/ml)	3.29 ± 0.12	3.66 ± 0.17
Liver lipids (mg/g liver)		
Total lipids	132.6 ± 10.8	90.6 ± 7.9**
Triglyceride	51.9 ± 0.9	45.0 ± 2.6*
Cholesterol	6.77 ± 0.45	4.32 ± 0.17**
Fecal lipids (mg/3d)		
Total lipids	71.4 ± 2.89	77.1 ± 2.48
Triglyceride	5.67 ± 0.32	7.50 ± 0.64*
Cholesterol	6.65 ± 0.35	6.72 ± 0.26

1. Each value is the mean ± SEM, n = 8.

2. Asterisks indicate significant differences as compared with mice fed a high-fat diet containing control IgY by Student’s t-test (*p < 0.05; **p < 0.01).

### Plasma parameters, liver and fecal lipids

The plasma triglyceride tended to be lower (p = 0.093) in the AY group than that of the CY group (Table 2). Liver triglyceride and cholesterol were significantly lower in the AY group than in the CY group. The fecal excretion of triglyceride was significantly higher in the AY-treated animals when compared with controls. Plasma glucose, insulin and TNF-α levels were not significantly changed between AY group and CY group.

## Discussion

To begin the search for alternative anti-obesity agents, we exploited a hen immunization model to produce Anti-lipase IgY, a novel antibody that recognizes porcine pancreatic lipase. The lipolytic activity was strongly inhibited in a concentration-dependent manner upon addition of Anti-lipase IgY when compared with Control IgY (Figure 1). These results suggest that Anti-lipase IgY reacts specifically with pancreatic lipase. Surprisingly, the IC_50_ of Anti-lipase IgY (0.49 μM) that elicits an anti-obesity effect is approximately half that of Orlistat, which has an IC _50_ of 0.96 μM [17]. Both of these treatments are in turn superior to the green tea polyphenol, (−)-epigallocatechin gallate (EGCG), which has an IC_50_ of 7.5 μM [3] accompanying an anti-obesity action in mice fed a high fat diet. Interestingly, as the effective dose of IgY is 0.2% (w/w) in animal study, it is the lowest value among functional food components such as EGCG [3,4].

Recently, the availability of lipase inhibitor agent for preventing metabolic syndrome has received much attention. Orlistat, an anti-obesity drug used in the clinic, is a potent and specific covalent inhibitor of digestive lipase [2,23], which leads to reduced intestinal absorption of lipolysis products. However, the use of Orlistat is frequently associated with gastrointestinal adverse effects, such as oily stools, diarrhea, cholelithiasis and cholestatic hepatitis [2,24]. But, judging from our observation and the data of liver lipids analysis, there is no side effect by the treatment of anti-lipase IgY as observed by Orlistat.

The effects of Anti-lipase IgY and Control IgY on obesity were evaluated in mice fed a high-fat diet containing 0.2% (w/w) IgY, respectively. Anti-lipase IgY supplementation significantly decreased adipose tissue weights and hepatic lipid levels and also significantly increased the fecal excretion of triglyceride compared with Control IgY feeding (Table 2). These results suggest an anti-obesity function of Anti-lipase IgY: it inhibits the hydrolysis of dietary fat in the small intestine and reduces intestinal absorption of dietary fat, which is then excreted into the feces.

To investigate the effect of anti-lipase IgY on the absorption of triglyceride in the diet, we analyzed fecal lipids contents. Previous study showed that lipase inhibition by synthetic lipase inhibitor Orlistat was extremely fast (half-inhibition time < 1 min) [25]. Moreover, in our preliminary study, single anti-lipase IgY administration tended to decrease in plasma triglyceride levels after single gavage of olive oil. These results indicate that the treatment with anti-lipase IgY for short term is effective for the increase in fecal triglyceride content. Thus, we performed fecal collection during day 7 through 9. On the other hand, it seemed to need longer time before the treatment with anti-lipase IgY become effective against the other measurements, such as adiposity and hepatic lipid accumulation. Thus, in this study, we investigated the effects of anti-lipase IgY treatment for longer period (35 days) on these parameters. Unfortunately, we have no data about a timing effect on fecal lipid excretion, and further investigation on it is important in future study. However, to our knowledge, there is no report about the tolerant to pancreatic lipase inhibitor, such as Orlistat. Moreover, Moreno *et al.* reported that dietary supplementation with peanuts shell extracts, which inhibit pancreatic lipase, significantly increased the excretion of fecal lipids in rats fed a high fat diet at 3 weeks of the study and the increase continued until the end of the study, 12 weeks [26]. In addition, Desmarchelier *et al.* reported that feeding mice a high fat Western diet containing cholesterol remarkably increased the excretion of fecal neutral sterol during day 4–11 of the experiment and the increase was kept until late in the experiments, day 74–81 [27]. These findings in the literatures suggest that diet-, nutrient- and food ingredient-stimulated enhancement of fecal lipid excretion observed in early experimental stage could be sustained even in late stage. Therefore, we determined fecal lipids during the relatively early stage and found a significant increase in the fecal triglyceride of mice treated with anti-lipase IgY, which is predicted to be kept until the late stage.

The aim of this study is to determine whether the anti-lipase IgY has the preventive effects on early obesity development. Obesity is defined as excessive fat accumulation [28]. Thus, in this study, we mainly investigated the effects of relatively short term treatment (35 days) with the anti-lipase IgY on the high-fat diet-induced development of white adipose tissues (WAT). Although the treatment with anti-lipase IgY didn’t affect body weight (tended to decrease but not significantly), we demonstrated that the anti-lipase IgY inhibited visceral WAT accumulation, and we evaluated that the anti-lipase IgY has an anti-obesity effect. A similar claim was made in the study done by Foucault AS et al. [29]. The reason why anti-lipase IgY didn’t affect body weight gain in this study may be due to the experimental period because circular triglyceride level in mice fed control HFD in this study is not higher than those in mice fed HFD for prolonged period in previous studies [30,31]. Therefore, further investigation is needed about the effects of anti-lipase IgY treatment for longer period on the body weight gain and obesity-induced metabolic disorders, in future. However, a specific pancreatic lipase inhibitor, Orlistat, has been used in clinics for the prevention of obesity [2], and Orlistat has been shown not only to prevent body weight gain but also to improve obesity-induced metabolic disorders, such as glucose intolerance and diabetes [32]. Our results showed that anti-lipase IgY has more potent inhibitory effect on the pancreatic lipase than Orlistat (IC50 values: 0.49 μM vs 0.96 μM) [17]. Thus, we think we can expect that anti-lipase IgY has useful effect on body weight gain and obesity-induced metabolic disorders in the prolonged treatment.

Taken all together, the data from our study show that Anti-lipase IgY is a promising new lead biologic agent for the development of functional foods and medicines expected to prevent and improve obesity.

## Competing interests

The authors declare that they have no competing interests.

## Authors’ contributions

Contribution of each author: S N designed the research and wrote the manuscript; M H conducted the research and wrote the manuscript; T A, R S and K U conducted the research; Y K, SV N, T G and M S analyzed the data. All authors read and approved the final manuscript.
